# A Preliminary Study on the Siphon Mechanism in Giraffe (*Giraffa camelopardalis*)

**DOI:** 10.3390/ani12233348

**Published:** 2022-11-29

**Authors:** Marna Suzanne van der Walt, Willem Daffue, Jacqueline Goedhals, Sean van der Merwe, Francois Deacon

**Affiliations:** 1 Department of Animal, Wildlife, and Grassland Sciences, Faculty of Natural and Agricultural Sciences, University of the Free State, Bloemfontein 9301, South Africa; 2 Kroonstad Dierehospitaal, Kroonstad 9499, South Africa; 3 Department of Anatomical Pathology, Faculty of Health Sciences, University of the Free State, National Health Laboratory Service, Bloemfontein 9301, South Africa; 4 Department of Mathematical Statistics and Actuarial Sciences, University of the Free State, Bloemfontein 9301, South Africa

**Keywords:** giraffe, siphon, cerebral blood supply, ostrich, dinosaur

## Abstract

**Simple Summary:**

Long-necked animals like giraffes face the challenge of adequately supplying blood to the brain. The heart of such tall animals must work harder to pump blood over a 2 m head-to-heart distance against the force of gravity compared to a short-necked animal. Does a mechanism exist to assist tall animals in overcoming this challenge? We found that there is a possibility of a mechanism that can enhance blood flow to the brain and a subsequent increase in oxygen supply, which will reduce the heart’s workload to pump blood over a 2 m distance in a Giraffe. In short-necked animals, the workload on the heart is less to supply blood to the brain adequately; therefore, no additional mechanism is necessary. We, however, suggest that a similar mechanism might have been functional in dinosaurs with extremely long necks to help with adequate blood supply to the brain by comparing relevant giraffe physiology and anatomy with existing data on paleontology.

**Abstract:**

Adult giraffes reach heights of 4.5 m with a heart-to-head distance of over 2 m, making cranial blood supply challenging. Ultrasound confirmed that the giraffe jugular vein collapses during head movement from ground level to fully erect, negating the possibility of a siphon mechanism in the neck. We showed that a short-length siphon structure over a simulated head-to-heart distance for a giraffe significantly influences flow in a collapsible tube. The siphon structure is determined according to brain case measurements. The short-length siphon structure in a shorter-necked ostrich showed no significant increase in flow. The shorter head-to-heart distance might be the reason for the lack of effect in ostriches. A siphon mechanism situated in the cranium is certainly possible, with a significant effect exerted on the amount of pressure the heart must generate to allow adequate cranial blood perfusion in a long-necked giraffe. The study validated that a cranial-bound siphon structure can operate and will be of significant value for adequate cranial blood perfusion in long-necked species such as giraffes and might also have existed in extinct species of long-necked dinosaurs.

## 1. Introduction

Effective circulation in long-necked animals is controversial, contentious and has been mentioned before by several scientists [1,2,3,4]. In fact, Ref. [5] discussed the lack of siphon effects in giraffes. However, research on the effect of gravity on anatomical structures such as the heart and blood vessels, and the subsequent effect thereof on blood pressure and cardiac workload of tall animals, must still be expanded in addition to the current literature. The vascularisation of giraffes is yet to be conclusively resolved. As described by [6], the earth’s gravitational force notably affects blood pressure. Three forces are described to exert an effect on the flow of a liquid in a tube structure: gravitational force, accelerative or kinetic energy force, and the force of viscosity or friction of the liquid. The circulation in mammals is described as a closed-tube system; therefore, the force of gravity hampers blood flow in the upward flow direction within the system [7]. Since the upward direction of blood flow in the closed vascular system is hampered by gravitational force, an energy source is essential to overcome resistance and power blood flow. Therefore, blood flow in the arteries is affected by gravity. The energy generated by the pumping of the heart powers the flow of blood [7,8,9,10,11]. Hence, the cardiac workload in larger animals will be greater to counteract the forces of gravity in the direction of blood toward the head and the body’s peripheral resistance to blood flow. A higher cardiac workload is essential to sustain higher blood pressure [12]. Ref. [7] proposed a siphon mechanism where energy is recovered from blood returned to the heart via the jugular vein, creating the energy that is necessary to fuel arterial flow against gravitational force along the neck of the giraffe without adding to the workload of the heart. For a siphon mechanism to be at work, there has to be a continuous flow of blood in the arterial and venous systems [13]. In humans sitting in an upright position, negative pressures are measured [6]. Therefore, the intracranial sinuses and the veins sustain negative pressures and consequently support an operational siphon. Even though studies showed that the jugular veins collapse under negative gravitational pressure, the intracranial venous sinus system has not been considered as an alternative for a siphon mechanism where circulation is not ceased due to collapse since it is enclosed by the rigid structure of the braincase where collapse is minimal [7,13,14,15,16,17,18]. Such extensive focus has been placed on the jugular veins that the importance and functionality of the vertebral veins in cerebral blood circulation have been largely ignored. Similarly, it is assumed that vertebral veins will mirror the phenomenon of collapsing under negative gravitational pressures. External structures and structures surrounding vertebral veins play a vital role in collapsibility [8,9,13,15,18]. Even though the siphon concept from the head to the heart has been extensively researched, cerebral functionality has been neglected. In a giraffe, a siphon positioned dorsal then caudal in the cranium can be of significant importance for blood outflow in the cranium. Functionally, brain circulation includes several mechanisms and commodities, including intracranial fluid pressure and the spatial effect on hydrostatic pressure [19]. The possibility of the intracranial venous sinuses as the only path in conjunction with the jugular vein unlocks the opportunity for a siphon mechanism to operate within the cranium of a giraffe. The constant flow requisite for the siphon is then governed by blood flow through intracranial venous sinuses and the collapsible jugular vein [15]. The amount of blood flow through the jugular and vertebral veins in the giraffe has not been comparatively measured. The average systemic blood pressure in humans is 50–150 mmHg [20,21].Flow through capillaries is constant within this pressure margin. Therefore, blood flow through the giraffe brain is not dependent on the pressure within this margin. Cerebral vascular resistance is the relationship between cerebrospinal fluid pressure and total pressure drop across the vascular bed. The difference in pressure between the capillary and venous pressure determines the gradient, and thus the resistance to flow, across the vascular bed. In the brain, intravascular pressure drops from mean arterial to venous pressure in large veins (sinuses) [21]. The resistance of a single arteriole is high. Still, the effective resistance of the capillary bed is low due to a large number of parallel capillaries branching from a single arteriole. If a cranial bound siphon exists, it can reduce cerebral venous pressure; therefore, the pressure gradient is reduced, aiding blood movement through the brain parenchyma. The simplest and easiest way for the pressure gradient between the arterial and venous sides to aid blood flow through the brain is by decreasing venous pressure, which is precisely what a siphon mechanism will do; this is why research into the vascularisation of the giraffe brain should be expanded and studied in more detail.

In the discussion of the possibility of a siphon mechanism in the cranium, the exceptionally developed anastomotic connection between the carotid artery and the vertebral artery in giraffes can all be part of the list of structures that. in conjunction. aid in the effective blood circulatory control in long-necked giraffes [22]. Ref. [23] commented that veins in the cranium of sauropod dinosaurs could be inhibited from characteristic collapse via a similar structure to the vertebral venous plexus. Moreover, in humans, Ref. [9] showed that the tough dura mater is divided to avoid cerebral vein collapse. Ref. [23] propose that sauropods may have had mechanisms, such as intracranial sinuses and dura mater, analogous to human ones in order to successfully manage cerebral venous collapse due to negative blood pressure in the brain. Is this a possible mechanism in long-necked individuals, such as giraffes and sauropod dinosaurs, as part of the mechanisms in place to handle negative cranial pressures? Several other authors have stated that a siphon mechanism is not operational in the giraffe [3,16,17,24,25]. One such argument is that giraffes have very high arterial blood pressure, which suggests an additional workload on the heart to push the blood column towards the brain for adequate perfusion. In support of this argument are the analyses done on blood pressure and body mass association, where blood pressure increases with an increase in mammal body mass [25]. However, when the blood pressures of house sparrows and ostriches were compared, the ostrich did not have significantly higher blood pressure, as would be expected for the long-necked ostrich where there is an additional workload on the heart working against gravity [25]. Birds are known to have higher blood pressure in comparison to mammals [26], and it was found that blood pressure is not related to body mass [25]. Therefore, the siphon mechanism cannot be refuted only by the presence of high arterial blood pressure.

A siphon mechanism in birds and sauropod dinosaurs is a definite possibility if circulating venous blood from the brain can be diverted into either the internal jugular veins or the intracranial venous sinuses or partially into both. This mechanism will ensure continuous blood flow, which is a prerequisite for a siphon to operate and makes sense if the sauropod and the giraffe drinking behaviour are considered [23]. However, in the giraffe, a basilar artery is rudimentary, but in dinosaurs and birds, the basilar artery is fully functional [27,28]. Additionally, future research should also aim to investigate if the basilar vein is present in dinosaurs. We have not observed a basilar vein in the giraffe. A study was done on thermoregulation in penguins (*Spheniscus demersus*) and stated that the function of the vertebral venous plexus is to reduce pressure at the spinal cord level as well as function as the primary pathway for cerebral venous blood return when erect [29]. [30] argued that dinosaurs, the evolutionary precursors of birds, cannot cope with the low pressures since numerous arteries and veins that run along the necks of dinosaurs and birds are entirely exposed and thus vulnerable to collapse.

In this study, we aimed to to the following.

Firstly, to illustrate the jugular vein reaction during the head movement from ground level to fully erect.

Secondly, to determine if continuous flow is possible in a collapsible tube resembling the length of a giraffe neck and also the length of an ostrich neck.

Lastly, to determine the effect of a cranial-bound siphon mechanism in giraffes and ostriches.

## 2. Materials and Methods

The study included five giraffe specimens and will test if a siphon is operating in the neck of giraffes and ostriches (n = 5). The possibility of a siphon operating in the cranium will also be investigated (UFS-AED2020/0083).

### 2.1. Experiment Part 1: Measurement of the Carotid Artery and Jugular Vein at Upright Posture

This part of the study and experiment was conducted on one male giraffe (*G. Camelopardalis*). The experiment was conducted on only one giraffe due to the following factors:

1. Giraffes are highly susceptible to capture-related death;
2. Restricted accessibility to highly sophisticated and expensive technological equipment to use in the field (none available for animal studies);
3. Limited human cardiac radiologists available to operate the human, technical equipment in the field with large herbivores.

**Figure 1:**
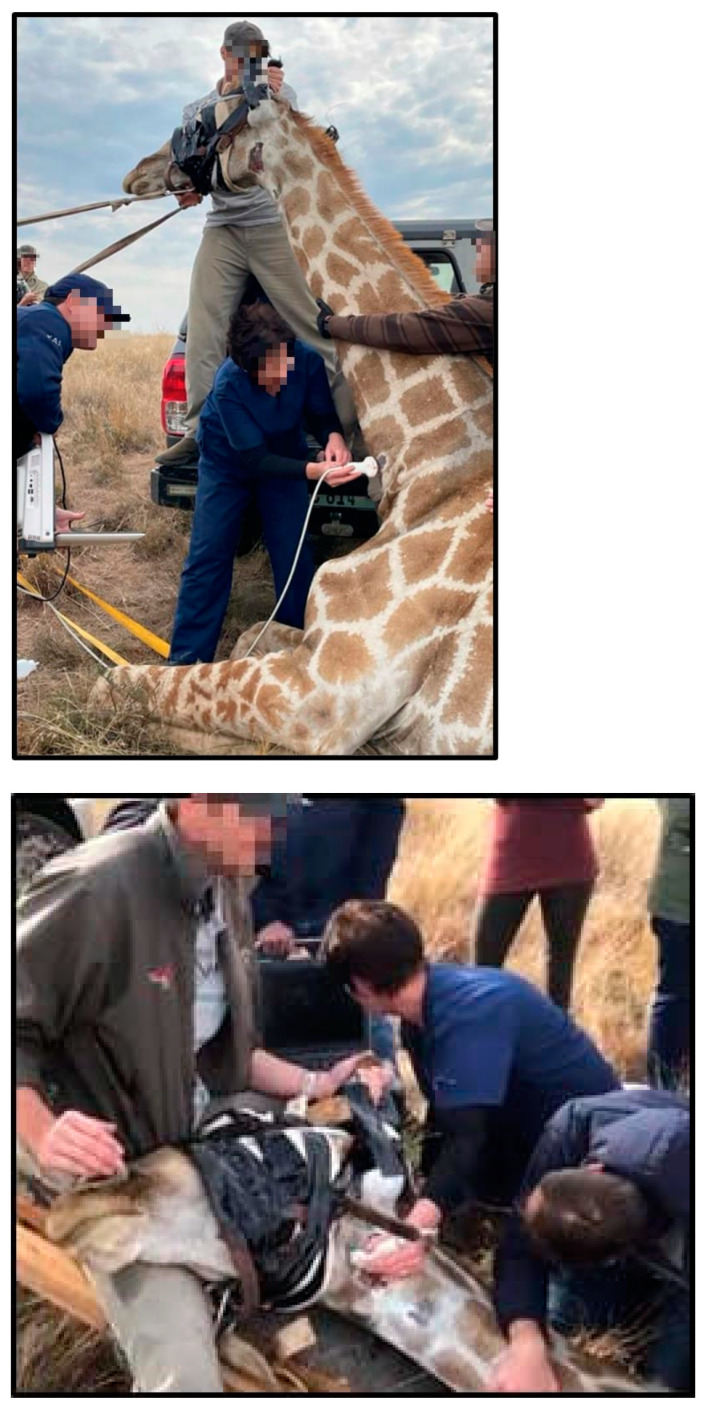
Sonar measurement of the jugular vein and carotid artery with head-down position, measured at the medial aspect of the neck in a distal position.

A Pneudart 389 dart gun was chemically used to immobilise a single male giraffe in the rump area. A 2 cc dart (Motsumi) with a 2.5” twin-port wire barbed needle was used. The dart was loaded with the captured drug A3080 (Thiafentanil). The dart contained 1 mL of A3080 (10 mg/mL) and 1 mL of sterile water. The dart must be filled to its total volume to prevent an unbalanced flight. A3080 is an opioid drug known as the immobilisation or knock-down drug [31]. Within 10 min, the characteristic high-trot behaviour of the effects of the captured drug was evident. The giraffe was roped down and kept on the ground. Immediately, post recumbency, the antagonistic drug M5050 (Diprenorphine) (1 mL M5050) was administered intravenously to allow regaining of consciousness, and improved breathing is evident within 20 s post-administration. This is essential as giraffes are extremely sensitive to immobilising drugs due to the drug’s adverse effects on blood pressure. Side effects of opioid medicines include respiratory depression, continuous excitement, inflexibility, rigid muscles, hyperthermia, hypertension and gut motility depression [31]. Failure to reverse the immobilising drugs can easily result in death [31,32]. After administering the antagonist drug, the giraffe regains full consciousness and is awake during measurements. The eyes were covered with a mask, and the ears were stuffed with cotton wool and taped to prevent stimulation via sound or sight from adding to the stress experienced by the animal due to the capture procedure. In order to conduct all the measurements, the giraffe was kept in sternal recumbency, and the head was moved to obtain an upright and downward position (Figure 1).

Measurements were conducted with a SonoScape S8 Exp Ultrasound device fitted with a 3.6–5 MHz convex probe. Blood vessels of the giraffe lay deep within the neck, and therefore a convex probe was used with a lower frequency to obtain deeper penetration. The areas selected for measurements were shaven clean, and coupling gel was applied to the shaven areas. The diameter of the proximal and distal common carotid artery and the internal jugular vein in a head-upright and head-down position was measured by applying the convex probe to the sites in the neck.

### 2.2. Experiment Part 2: Braincase Measurements

This part of the study again allowed scientists to obtain access to a giraffe (n = 5) and ostrich (n = 5) skulls. The braincase was measured in the skulls of n = 5 adult giraffes (n = 2 female, n = 3 male), n = 5 adult ostriches (n = 2 female, n = 3 male) and n = 1 sauropod dinosaur (*Sarmientosaurus*) by using a calliper (RS Pro Imperial, Metric vernier calliper). The cranium was thus measured from top to bottom to determine the possible length of a siphon mechanism that might operate in the cranium. The blood flow increase percentage to the brain was calculated using the following calculation:

braincase measurement (mm) × 100 = percentage increase blood flow and thus O_2_ delivery neck length measurement (mm) (1)

### 2.3. Experiment Part 3: Blood Flow Simulation

**Figure 2:**
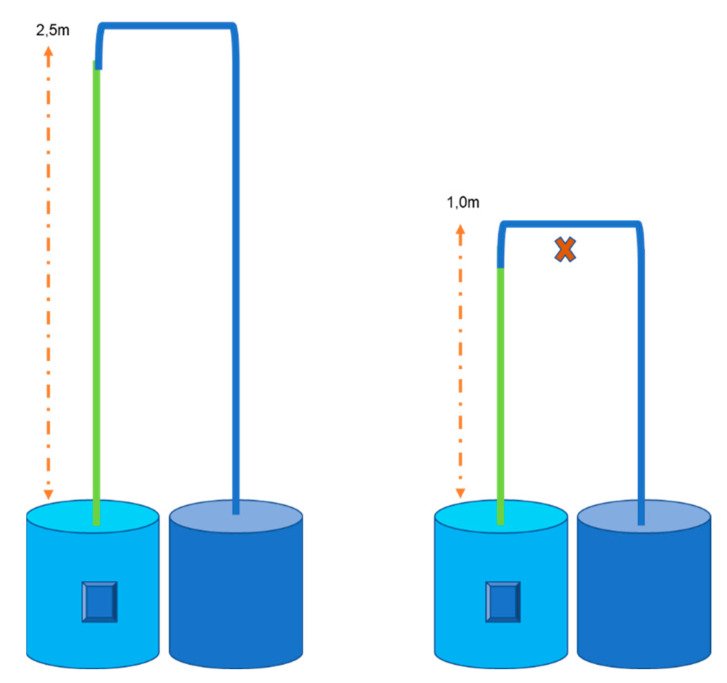
Tubing constructed representing the giraffe neck (**left**) and the ostrich neck (**right**) schematically, with the red cross indicating the brain.

**Figure 3:**
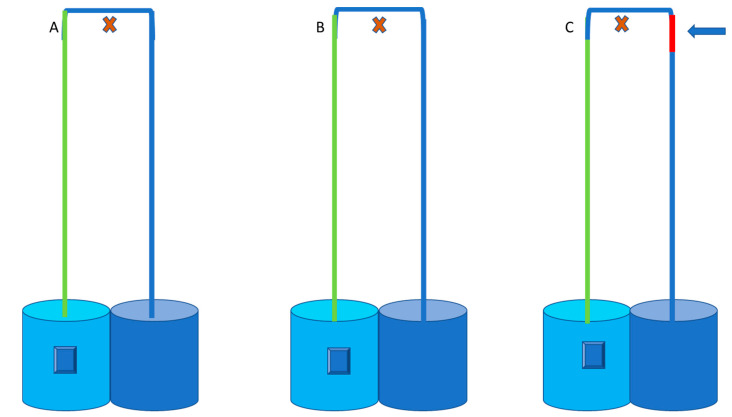
Schematic presentation of the three variations with non-collapsible (**A**), collapsible (**B**) and collapsible tubing with cranial siphon add-in (**C**) as indicated by the blue arrow and the red cross indicating the brain.

An inverted U-Shaped simulation experiment of blood circulation from the heart to the head and back to the heart was conducted against an outdoor wall. An experiment conducted by [23]) to test if a siphon can operate in the sauropod dinosaurs will be used as a guide to fit the neck measurements of a giraffe (2.0 m) and ostrich (1 m). The siphon mechanism was tested by simulating blood flow from the heart via the carotid artery to the brain and returning blood from the brain via the jugular vein back to the heart. The tubing represents arteries and veins in a giraffe and ostrich with the neck raised from ground level to an upright posture. Plastic PVC tubing (10 mm diameter) was constructed against an outside wall (Figure 2). A tape measure was used to measure 1 m and 2 m height from ground level, indicated by the orange dotted lines with arrows, to represent neck length, i.e., distance from the heart (ground level) to the head, in ostrich and giraffe, respectively. A 10-litre bucket was filled with water (temperature of 25 °C), and a submersible electric pump (Grech HJ-542) was fitted to the plastic piping and placed at the bottom of the bucket, providing constant pressure. The turquoise bucket with the pump, indicated by a blue block, represented the heart pumping blood towards the brain, indicated by the red cross. The green line indicates the carotid artery. Three variations of tubing were used to represent the jugular vein section (blue line), to test blood flow in (1) non-collapsible PVC tubing, (2) collapsible tubing, (3) collapsible tubing with a siphon section add-in, indicated by a red line, at cranial level (Figure 3). The siphon section add-in was 60 mm long for the ostrich and 100 mm for the giraffe. A bucket with markings representing the jugular vein was placed at ground level at the outlet end of the tube to measure water pumped through the inverted u-tube structure. The pump was started and ran for 1 min, whereafter it was switched off. Water flow was measured for 1 min with the amount of water in the receiving bucket measured. Water flow measurements for both the ostrich and the giraffe were repeated ten times for each of the three variations and were calculated in litres per second.

### 2.4. Statistics

The significance level chosen is 0.05. We used the standard analysis of variance (ANOVA) approach to determine whether the groups differ on average. We used the Kruskal–Wallis approach to check whether the groups vary in a more generic sense of systematically larger or smaller values in one group versus the other.

## 3. Results

### 3.1. Experiment Part 1: Measurement of the Carotid Artery and Jugular Vein in an Upright Posture

**Figure 4:**
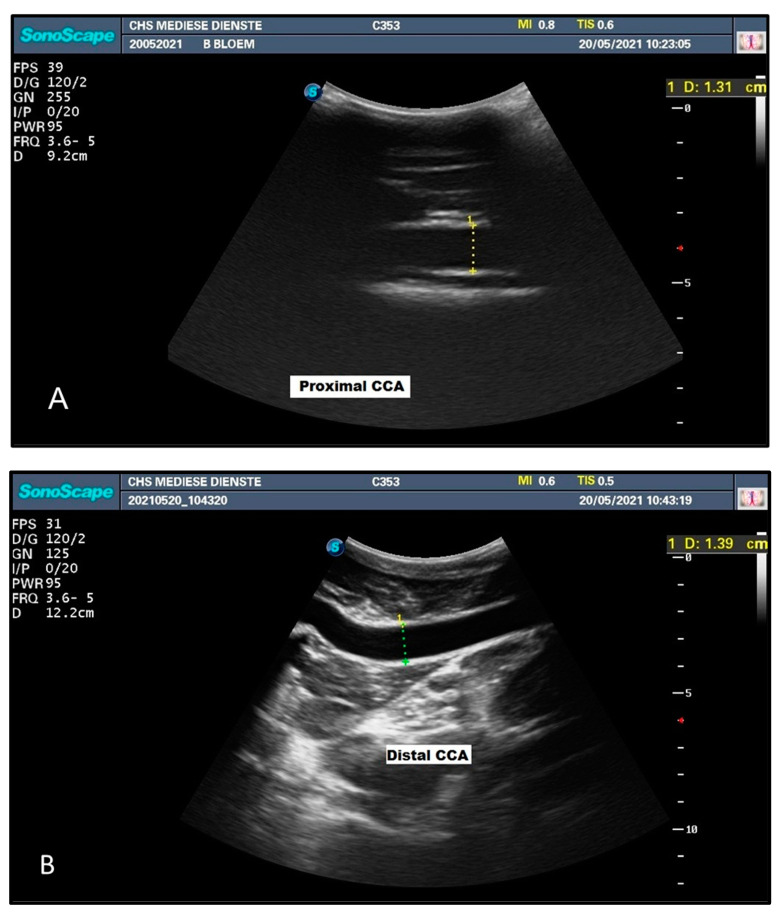

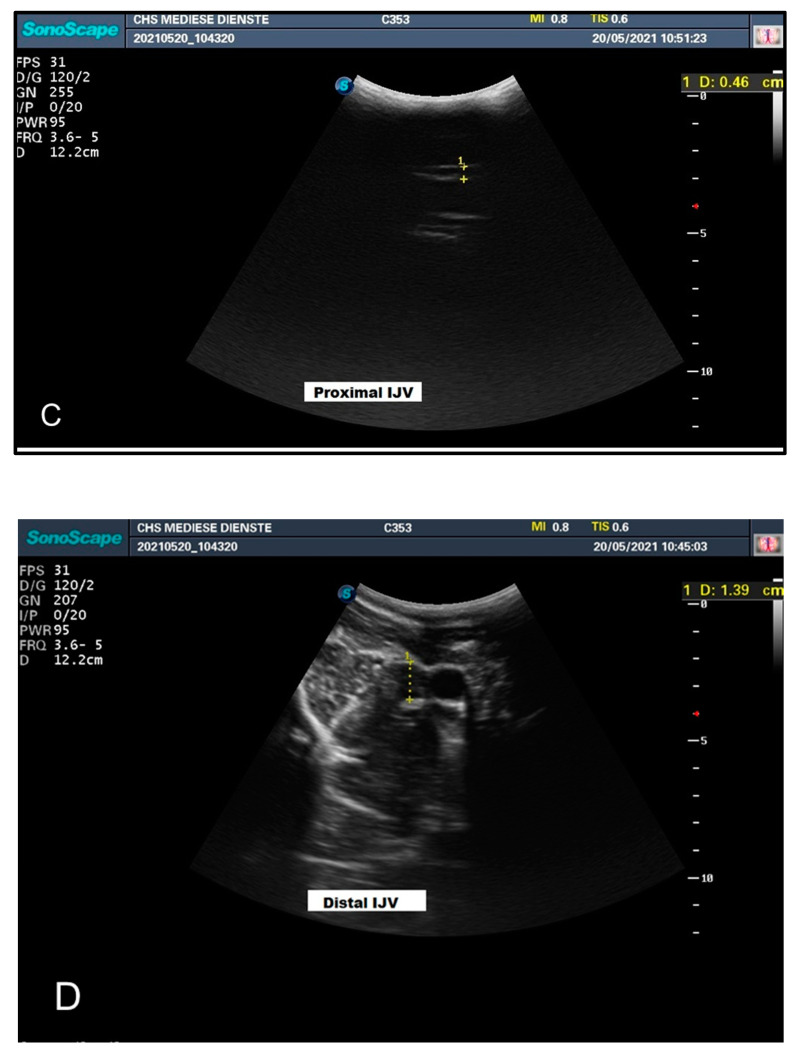

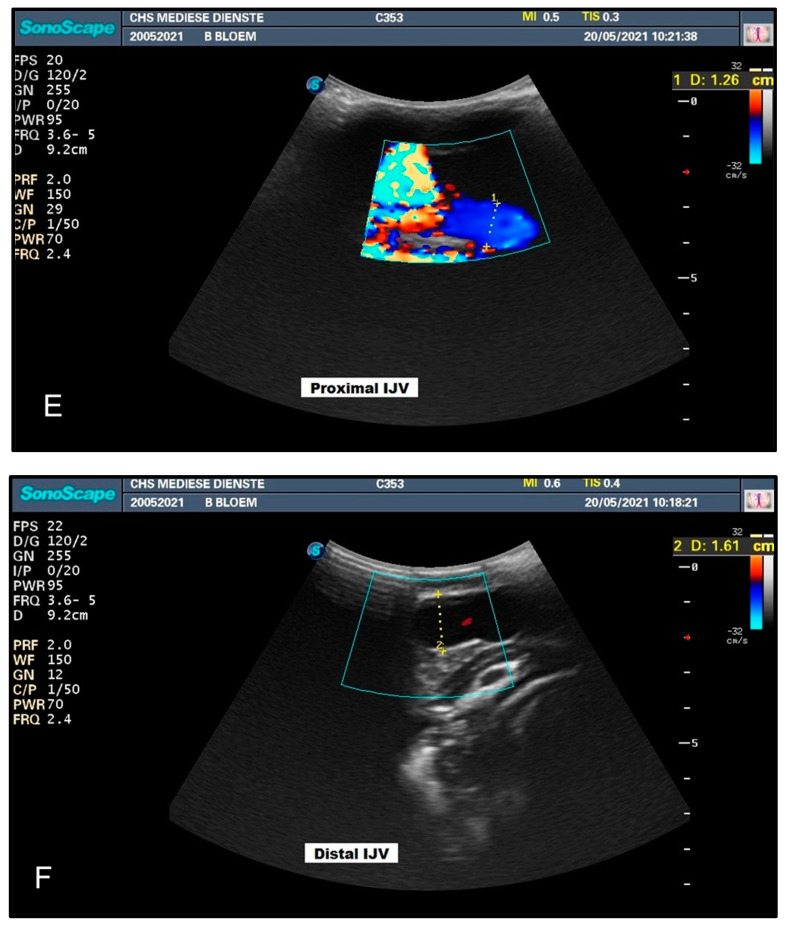
Diameter of the proximal (**A**) common carotid artery (CCA) with head lowered and distal (**B**) common carotid artery with head in the upright position. Diameter of proximal (**C**) internal jugular vein (IJV) and distal (**D**) internal jugular vein in an upright head position. Diameter of proximal (**E**) internal jugular vein (IJV) and distal (**F**) internal jugular vein in a head-lowered position.

A young giraffe bull aged 7–8 years was skillfully and successfully immobilised with a trained and experienced giraffe capture team for the cardiology radiologists to perform the sonar. This can be considered as a preliminary pilot study to investigate in more detail in the future. The duplex ultrasound examination with the ultrasound device measured the dimensions as indicated in the table (Table 1) and figure (Figure 4) below. The proximal and distal measurements of the common carotid artery for both the head-up and head-down positions were relatively similar, showing no signs of size change or collapse. The proximal and distal measurements of the internal jugular vein, however, are notably further apart in both the head-up and head-down positions, indicating the effect of head position on size, and thus on flow, in the internal jugular vein. In the head-up position, the proximal and distal measurements of the internal jugular vein are remarkably far apart, with the proximal measurement markedly smaller than the distal measurement, indicating collapse at this position.

### 3.2. Experiment Part 2: Braincase Measurements

**Figure 5:**
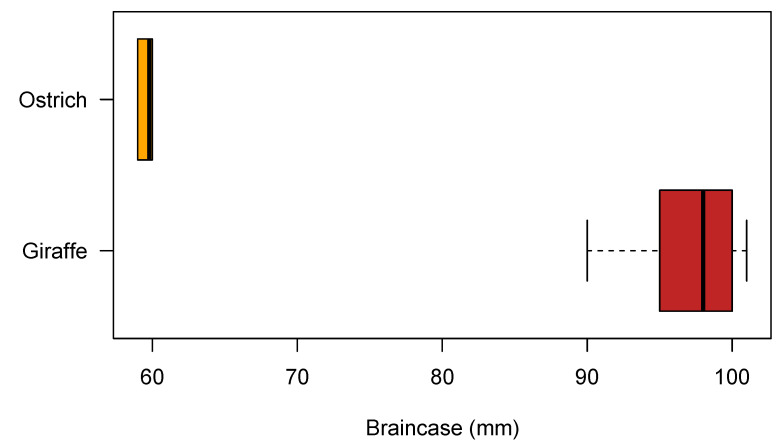
The box plot illustrates the braincase measurements for both the giraffe and ostrich.

Skull samples were retrieved for giraffes (n = 5) and ostriches (n = 5) to measure the brain case of each skull (Figure 5). A 3D endocast figure was available to measure the braincase for a sauropod dinosaur, Dinosuaria: Sauropoda, Sarmientosaurus musacchioi (n = 1) [33]. The neck length of Sarmientosaurus is unknown, but sauropod necks ranged from 9–12 m in length [1]. We used average neck lengths for giraffes to calculate the blood flow increase percentage.

In giraffes, blood flow to the brain will be enhanced by 5 per cent and in ostriches by 6 per cent, with the existence of a cranial-bound siphon mechanism. In sauropods, the estimation of blood flow to the brain will be enhanced from 0.7 per cent in a sauropod with a 9 m-long neck, i.e., head to heart distance, to 0.6 per cent in a sauropod with a 12-m long neck.

### 3.3. Experiment 3: Blood Flow Simulation

Blood flow simulations for ostriches (Table 2A) and giraffes (Table 2B) were obtained and expressed in litres per second. Density plot of flow simulation values for each measurement (L/s) (collapsible tube, non-collapsible tube, collapsible tube with siphon) for each species (Figure S1).

**Figure 6:**
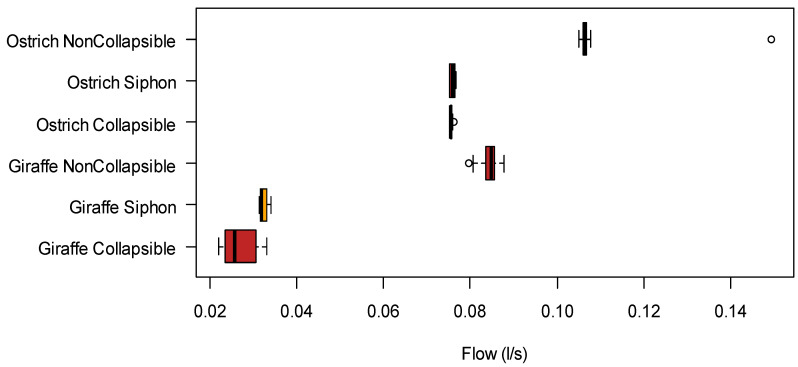
Distribution of flow simulation values of each measurement (L/s) (collapsible tube, non-collapsible tube, collapsible tube with siphon) for each species, with outliers included as indicated by white circle.

A box plot visually illustrated the distribution of the values for each measurement for both ostriches and giraffes (Figure 6) and is supported by the statistical tests.

The analysis of the variance approach indicated that differences between species, measures, and species–measures interaction were significantly different (Table 3). The normality test of residuals (Shapiro–Wilk) showed a *p*-value of <0.05, however.

The Kruskal–Wallis, a non-parametric test, was implemented to test for group differences. The *p*-value of *p* < 0.05 showed a significant difference between groups.

The Pairwise Wilcox Test was implemented to investigate non-parametric differences between groups. Table 4 shows the differences between groups. All the groups showed significant differences between groups except for the ostrich group, where the siphon data were not significantly different from the collapsible tube data. This might be due to the short cranial structure not having a notable effect on the relatively more straightforward neck of the ostrich, with the effect only notable at a longer neck length, such as in the giraffe’s case.

## 4. Discussion

Long-necked giraffes experience several physiological challenges due to their extraordinary physique. Detailed tests on captive giraffes showed that these animals have a very high blood pressure of approximately 200–400 mmHg [24,34,35]. At this pressure, specific key physiological issues arise. For instance, the heart must pump blood to the brain against gravitational force and would encounter vascular friction towards the brain, located nearly 2 m above the heart. Movement of the head from ground level to fully erect should result in fainting, with gravity pulling blood away from the brain towards the heart. Due to the force of gravity, the giraffe’s blood pressure in standing upright position is similar to the blood pressure of other mammals [5,8].

Additionally, when the head is moved from fully erect to ground level, a large blood column rushes to the brain along the gravitational gradient, which should result in brain damage. The concerns of several researchers discussing a siphon model in long-necked animals is that only the vessels in the cranium, intracranial venous sinuses and spinal cord will not collapse due to the structural support of the structure of the cranium, the spinal cord and the dura mater [36,37]. A study conducted on humans [13,38] showed that when the blood leaves the protective structures of the braincase, the jugular veins collapse under the opposing pressures of gravity. This is due to the increased opposing forces in the cerebral veins, with the effect of a long distance between the head and heart of a human with an elevated stance on the head. The alternative route is the vertebral veins, protected from collapse by the vertebral structures and ligaments around them [13,18]. In the giraffe, we observed no anastomotic connection to the vertebral veins, negating the possibility of blood moving to the vertebral veins. This alternative route is then utilised. Therefore, a siphon mechanism is operative in humans sitting in the upright position, with cooperative pressures in the vasculature and sub-atmospheric pressure in the cerebral spinal fluid, preventing vascular collapse [36]. Veins and arteries in the brain are similarly protected in mammals by the cerebral bony structures and the cerebrospinal fluid pressure in the brain [13,18].

Similarly, the blood can be directed towards the vertebral veins in mammals. However, this cannot be true for species like the giraffe. The giraffe has no basilar artery [27,28], and we have not observed a basilar vein. The rudimentary basilar, therefore, cannot fulfil the role of an alternative route for supplying blood to the circle of Willis [39,40]. We suggest that future research should specifically confirm the absence of the basilar vein in giraffes. One argument against the siphon mechanism arises from the exceptionally high blood pressure of long-necked animals such as giraffes. The argument specified that high blood pressure forces blood from the heart to the head without needing a siphon [24,25]. In a study conducted on blood pressure in mammals, blood pressure scales with body mass [25]. Also, for a siphon mechanism to be operative, continuous flow is a prerequisite [13]. Similar to other scientists [7,14,15,16,17], we showed through ultrasound and by mimicking the blood flow with collapsible tubing that the jugular veins of giraffe do collapse when the head is moved from ground level to fully erect. The jugular venous collapse was also confirmed by several studies [2,14,15,41]. They concluded that the jugular veins had a very small lumen through which blood leaks and that the cranial part of the jugular veins retains a blood column, resulting in a small cross-sectional area in the cranial region. This is, however, because more prominent veins from the neck area join the jugular vein below this point and not due to the influence of pressure differences in the jugular vein.

Ref. [5] showed through ultrasound that a steady flow resumes in the collapsed jugular vein but negates the existence of a siphon because the vascular resistance in the jugular vein countervails negative pressure due to gravitation. Furthermore, Ref. [5] showed that the cranial part of the jugular vein also collapsed at the head-up position, which is different from this study’s findings. A possible influence might be due to the substantial effect of the Alpha-2 agonist (Medetomidine) on blood pressure [31], which was used in conjunction with the opioid drug in the study of [5]. The opioid effects were reversed in both the present study and by [5], but they did not reverse the alpha-2 agonist. Additionally, jugular venous pressures vary extensively in different studies [4,5,24,42], necessitating further research. We agree with the findings of [5] that the siphoning effect, along the entire length of the neck, cannot operate in the giraffe; the siphoning effect, which would be even more elaborate in a 12 m sauropod dinosaur, would also not be operational.

Several researchers’ concerns about vessel collapse in a siphon mechanism are negated in vessels within the cranium and intracranial venous sinuses. Vessel collapse does not occur due to the structural support of the structure of the cranium, the spinal cord, and the pressures of the cerebrospinal fluid [36,37]. Veins and arteries in the brain are equally protected in mammals by the cerebral bony structures and the cerebrospinal fluid pressure in the brain [13,18]. A siphon operating in the head to perfuse the brain is thus possible. Therefore, if a siphon is cranium-bound, the venous pressure in the brain and, subsequently, the intracranial pressure will be lower, resulting in enhanced brain perfusion. This can possibly add to other mechanisms to allow adequate circulation in long-necked animals. [13,38] proposed that the siphon mechanism has always been investigated in total, but restriction to cerebral circulation has not received adequate attention. Articles favouring the siphon principle all concluded that extensive amounts of energy would be saved with an operative siphon mechanism [8,23,36,43,44]. A siphon will lessen the amount of work that needs to be exerted by the heart to circulate blood across the considerable distance between the head and heart of a giraffe [30]. What influence would a siphon mechanism exert if it only existed in the cranium? How much blood is needed to adequately support cranial function in giraffes, long-necked birds such as the ostrich and ultimately sauropod dinosaurs?

In contrast to earlier studies, Ref. [45], in a review of the cardiovascular system of giraffes, summarised that the giraffe heart is, in several aspects, not different in comparison to other mammals. Although it is well documented that the structure of the heart of mammals is marcroscopically and microscopically different from that of currently living reptiles, future studies can also investigate direct similarities between their hearts and those of sauropods. The giraffe heart mass is within the limits of a typical mammalian heart [5,34,46]. Furthermore, the giraffe heart also has typical mammalian ventricular wall stress [25,46]. The thick walls and small left ventricular lumen, together with a higher tempo for the same cardiac output under very high pressure and the shape of the heart, are the features enabling the normal-sized giraffe heart to generate increased blood pressure [45]. The highly effective, smaller-than-expected giraffe heart, generating extreme pressures for efficient blood circulation, can benefit from the most minor decrease in energy to pump blood to the head. We have shown through simulations that in a collapsible tube, flow is significantly influenced by a short-length siphon structure over a simulated head-to-heart distance for a giraffe, with the length of the siphon structure determined according to brain case measurements. However, the short-length siphon structure in a shorter-necked ostrich did not show significant value. The shorter head-to-heart distance might be the reason for the lack of effect in the ostrich. Ostriches form part of the order Struthioniformes, described as one of the earliest groups descending from dinosaurs; they are thus classified as the oldest bird group [47]. Struthioniformes have notably small endocranial volumes indicating a less metabolically active brain [48,49]. The effectiveness of a small siphon in the cranium is also influenced by the amount of blood needed to adequately perfuse the brain and thus provide enough energy according to the metabolic activeness of the brain in a specific species [50](Hu et al., 2018). If less blood is needed due to a less metabolically active brain, the pressure generated by the heart is lower, with correspondingly lower energy usage. Higher metabolically active brains are associated with mammals [49,51] and decreased metabolic activity in the brains of the ostrich [49,51], and sauropod dinosaurs are a valuable route to investigate [51,52,53]. The mechanisms operating in giraffes can subsequently be extrapolated to mechanisms that might have been operative in sauropod dinosaurs [54], with a head-to-heart distance of over 9m [23]. The brain of dinosaurs is described as reptile-like, suggesting metabolically less active brains compared to birds and even less active ones in mammals [33,51]. Similar to ostriches, due to the less active brain in dinosaurs, less blood is needed to perfuse the brain adequately. The heart of a giraffe with a small intraventricular cavity and a thick ventricular wall and high tempo, producing significantly high arterial pressures [45], lead to suggestions by [23,46] that in long-necked dinosaurs, a heart similar to a giraffe heart, generating very high arterial pressures without being out of proportion in mass, is a possibility. With a smaller-than-expected efficient heart and the additional help of a cranial bound siphon, brain perfusion can be successfully executed in extremely long-necked sauropod dinosaurs.

A cranial-bound siphon mechanism will undoubtedly significantly affect the amount of pressure the heart must generate to allow adequate blood perfusion to the brain of the long-necked giraffe. As previously stated, we believe that no single mechanism is responsible for the physiological challenges giraffes face. The cranial-bound siphon structure, a smaller-than-expected, highly efficient heart [25,46,55], carotid-vertebral anastomotic connection [28] and a carotid rete structure [39,44,56] all contribute to meet physiological challenges originating from a unique body shape and size in giraffe. A unison of mechanisms in combination allows the giraffe to function effectively. Therefore, this tiny percentage increase in the effectivity of blood flow and subsequent increase in oxygen delivery efficiency due to the mechanism of a cranial-bound siphon adds to the features that allow the successful existence of this long-necked mammal.

## 5. Conclusions

While numerous studies have investigated single mechanisms that might assist long-necked animals, especially giraffes, to face the physiological challenges due to their unique build, we believe that a group of mechanisms work in unison to enable efficient cerebral blood supply when the head is in the upright position. This study discusses another mechanism that adds to the group of mechanisms allowing the giraffes to overcome the challenge of a long head-to-heart distance. Our study also allows future research to determine if some of these mechanisms were also functional in dinosaurs with extreme neck lengths and to shed light on the intricate vascularisation of the giraffe brain and how it functions to support long-necked animals.

## Figures and Tables

**Table 1:** Diameter of the proximal and distal common carotid artery (CCA) and the internal jugular vein (IJV) in a head-upright and head-lowered position.

Head Position	Diameter (mm) CCA		Diameter (mm) IJV	
	Proximal	Distal	Proximal	Distal
**Head Up**	13.8	13.9	4.6	13.9
**Head Down**	13.1	13.3	12.6	16.1

**Table 2:** (**A**). Flow simulations for an ostrich in a collapsible, non-collapsible, and collapsible tube with a siphon structure. (**B**). Flow simulations for giraffes in a collapsible, non-collapsible, and collapsible tube with a siphon structure.

(A)			
	Collapsible Tube (L/s)	Non-Collapsible Tube (L/s)	Collapsible Tube with Siphon (L/s)
1	0.075741182	0.149476831	0.075675676
2	0.076294278	0.106253795	0.076427558
3	0.075390415	0.106415324	0.075692042
4	0.075536851	0.105389943	0.076611579
5	0.075374179	0.104963263	0.076444250
6	0.075569470	0.105948237	0.076004343
7	0.076053890	0.106788711	0.076227812
8	0.075520552	0.105932203	0.075091182
9	0.075447295	0.107874865	0.075163750
10	0.075520552	0.106756138	0.075309306
**(B)**			
	**Collapsible Tube** **(L/s)**	**Non-Collapsible** **Tube (L/s)**	**Collapsible Tube** **with Siphon (L/s)**
1	0.021934635	0.085543199	0.031357792
2	0.025544648	0.083542189	0.031535793
3	0.025624131	0.080673044	0.033929524
4	0.025393601	0.084899939	0.032800712
5	0.021890049	0.079708495	0.033695966
6	0.029674848	0.087774295	0.033103187
7	0.030685604	0.085345038	0.031206812
8	0.033103187	0.083642012	0.031826862
9	0.023430178	0.084951456	0.031783509
10	0.031022868	0.085637387	0.031544320

**Table 3:** ANOVA summary of species, measures and species: measures.

	Df	Sumsq	Meansq	Statistic	*p* Value
Species	1	0.024	0.024	669.655	≈0
Measure	2	0.027	0.013	380.667	≈0
Species: Measure	2	0.001	0.001	19.5570	≈0

**Table 4:** Non-parametrical differences between groups are illustrated utilising the Pairwise Wilcox Test.

Group 1	Group 2	*p* Value
Giraffe Non-Collapsible	Giraffe Collapsible	0.000
Giraffe Siphon	Giraffe Collapsible	0.003
Giraffe Siphon	Giraffe Non-Collapsible	0.000
Ostrich Collapsible	Giraffe Collapsible	0.001
Ostrich Non-Collapsible	Giraffe Non-Collapsible	0.000
Ostrich Non-Collapsible	Ostrich Collapsible	0.001
Ostrich Siphon	Ostrich Collapsible	0.427
Ostrich Siphon	Ostrich Non-Collapsible	0.000

## Data Availability

The data and code are available at https://www.doi.org/10.38140/ufs.19947713 (accessed 28 November 2022).

## Supplementary Materials

The following supporting information can be downloaded at: https://www.mdpi.com/article/10.3390/ani12233348/s1, Figure S1: Density plot of flow simulation values for each measurement (L/s) (collapsible tube, non-collapsible tube, collapsible tube with siphon) for each species.
