# Supplementary material for: A Preliminary Study on the Siphon Mechanism in Giraffe (Giraffa camelopardalis)
Source: Animals (Basel). 2022 Nov 29;12(23):3348. doi: 10.3390/ani12233348 (PMC9740075; doi:10.3390/ani12233348)
Supplement: Supplementary file 1 [file animals-12-03348-s001.zip › animals-2047959-supplementary.pdf]

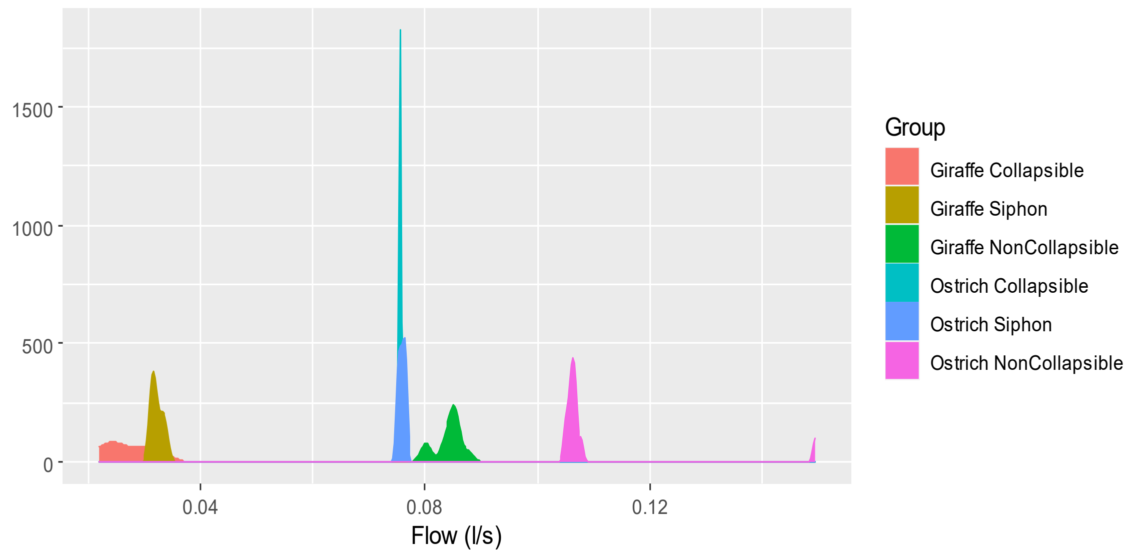

Figure S1. Density plot of flow simulation values for each measurement (l/s) (collapsible tube, non-collapsible tube, collapsible tube with siphon) for each species.
